# Why cannot a β-lactamase gene be detected using an efficient molecular diagnostic method?

**DOI:** 10.12669/pjms.325.9837

**Published:** 2016

**Authors:** Kwang Seung Park, Jung Hun Lee, Moonhee Park, Asad Mustafa Karim, Sang Hee Lee

**Affiliations:** 1 Kwang Seung Park, National Leading Research Laboratory of Drug Resistance Proteomics, Department of Biological Sciences, Myongji University, Yongin, Republic of Korea; 2 Jung Hun Lee, National Leading Research Laboratory of Drug Resistance Proteomics, Department of Biological Sciences, Myongji University, Yongin, Republic of Korea; 3 Moonhee Park, Faculty of DNA Analysis Division, Seoul institute, National Forensic Service, Seoul, Republic of Korea. National Leading Research Laboratory of Drug Resistance Proteomics, Department of Biological Sciences, Myongji University, Yongin, Republic of Korea; 4 Asad Mustafa Karim, National Leading Research Laboratory of Drug Resistance Proteomics, Department of Biological Sciences, Myongji University, Yongin, Republic of Korea; 5 Sang Hee Lee, National Leading Research Laboratory of Drug Resistance Proteomics, Department of Biological Sciences, Myongji University, Yongin, Republic of Korea

**Keywords:** β-Lactamase (*bla*) gene, Large-scale detection, Molecular diagnosis, Minimizing antibiotic resistance

## Abstract

**Objective:**

Fast detection of β-lactamase (*bla*) genes can minimize the spread of antibiotic resistance. Although several molecular diagnostic methods have been developed to detect limited *bla* gene types, these methods have significant limitations, such as their failure to detect almost all clinically available *bla* genes. We have evaluated a further refinement of our fast and accurate molecular method, developed to overcome these limitations, using clinical isolates.

**Methods:**

We have recently developed the efficient large-scale *bla* detection method (_large-scale_*bla*Finder) that can detect *bla* gene types including almost all clinically available 1,352 *bla* genes with perfect specificity and sensitivity. Using this method, we have evaluated a further refinement of this method using clinical isolates provided by International Health Management Associates, Inc. (Schaumburg, Illinois, USA). Results were interpreted in a blinded manner by researchers who did not know any information on *bla* genes harbored by these isolates.

**Results:**

With only one exception, the _large-scale_*bla*Finder detected all *bla* genes identified by the provider using microarray and multiplex PCR. In one of the *Escherichia coli* test isolates, a *bla*_DHA-1_ gene was detected using the multiplex PCR assay but it was not detected using the _large-scale_*bla*Finder.

**Conclusion:**

The truncation of a *bla*_DHA-1_ gene is an important reason for an efficient molecular diagnostic method (_large-scale_*bla*Finder) not to detect the *bla* gene.

## INTRODUCTION

The development of fast and accurate diagnostic methods to detect antibiotic resistance genes is needed to minimise antibiotic resistance.^1^ β-Lactam antibiotics are some of the most successful drugs used for the treatment of bacterial infections and represent roughly 65% of the total world market for antibiotics.^1^ Therefore, resistance to β-lactam antibiotics through the acquisition of genes that encode β-lactamases is one of the most serious problems in Gram-negative pathogenic bacteria. To date several molecular diagnostic methods of *bla* gene typing have been developed to detect the existence of β-lactamase (*bla*) gene(s) in clinical isolates.^2^-^8^ These methods can detect only some (limited) *bla* genes. Because these methods cannot detect *bla* gene types including almost all clinically available *bla* genes, they cannot perfectly explain the results of the culture-based phenotypic tests.^9^

This is a big problem in studying β-lactam resistance, as β-lactam resistance can increase due to inappropriate β-lactam use. To solve this problem, we have recently developed the efficient large-scale *bla* detection method (_large-scale_*bla*Finder) that can detect *bla* gene types including almost all clinically available 1,352 *bla* genes with perfect specificity and sensitivity.^9^

## METHODS

**Fig.1:**
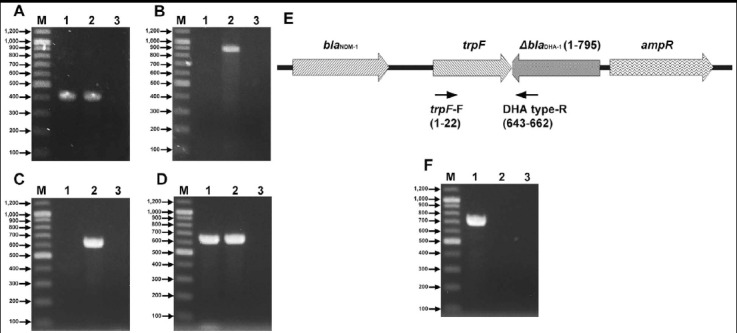
PCR assays to detect a truncated *bla*_DHA-1_ gene using an *Escherichia coli* test isolate (lane 1 of each Figure), *E. coli* E07-10537 (a *bla*_DHA-1_ positive isolate; lane 2 of each Figure), and a *bla*_DHA-1_ negative Providencia stuartii isolate (lane 3 of each Figure). (A) Simplex PCR assays using a primer pair (DHAMF and DHAMR) used by the method of Perez-Perez and Hanson (1). Two same bands (405 bp) were detected in the *E. coli* test isolate and *E. coli* E07-10537. (B) Simplex PCR assays using a primer pair (DHA(AmpC-2) type-F and DHA(AmpC-2) type-R) used by the _large-scale_*bla*Finder. Only one band (881 bp) was shown in *E. coli* E07-10537. (C) Simplex PCR assays using a primer pair (DHAMF and DHA(AmpC-2) type-R). Only one b and (642 bp) was detected in *E. coli* E07-10537. (D) Simplex PCR assays using a primer pair (DHA(AmpC-2) type-F and DHAMR). Two same bands (644 bp) were detected in the *E. coli* test isolate and *E. coli* E07-10537. (E) Schematic representation of the DNA sequences surrounding a truncated *bla*_DHA-1_ gene (Δ*bla*_DHA-1_) in *E. coli* 271 (Ho et al. (10)) and a newly designed primer pair. Each nucleotide position of Δ*bla*_DHA-1_ and each primer were shown in parenthesis. 345 bp (position: 796 to 1140) of *bla*_DHA-1_ sequence were missing at 3’ end. (F) Simplex PCR assays using a newly designed primer pair (trpF-F and DHA type-R). Only one band (734 bp) was shown in the *E. coli* test isolate. M1 (size marker), 100 bp DNA ladder (Biosesang, Korea).

We have evaluated a further refinement of this method using clinical isolates provided by International Health Management Associates, Inc. (Schaumburg, Illinois, USA), using the _large-scale_*bla*Finder method.^9^ Results were interpreted in a blinded manner by researchers who did not know any information on *bla* genes harbored by these isolates. With only one exception, the _large-scale_*bla*Finder detected all *bla* genes identified by the provider using microarray (Check-MDR CT101, Check-Points B.V., Wageningen, the Netherlands) and multiplex PCR.^2^ In one of the *Escherichia coli* test isolates, a *bla*_DHA-1_ gene was detected using the multiplex PCR assay designed by Perez-Perez and Hanson^1^ but it was not detected using the _large-scale_*bla*Finder (Fig.1A and B).

To resolve this issue, simplex PCR assays^9^ were performed for the detection of *bla*_DHA-1_ gene using the *Escherichia coli* test isolate, *E. coli* E07-10537,^9^ and a *bla*_DHA-1_ negative *Providencia stuartii* isolate.

## RESULTS

Interestingly, in the *E. coli* test isolate, no band was detected using the reverse primer (DHA(AmpC-2) type-R)^9^ used by the _large-scale_*bla*Finder (Fig.1C and D). The nucleotide position of the primer pair used by Perez-Perez and Hanson^2^ is 258-662. However, the nucleotide position of the primer pair used by the _large-scale_*bla*Finder is 19-899. The results suggest that there is a truncated *bla*_DHA-1_ (Δ*bla*_DHA-1_) lacking a 3’ (or 5’) end sequence in the *E. coli* test isolate.

## DISCUSSION

The previous study showed a Δ*bla*_DHA-1_ lacking a 3’ end sequence (Fig.1E).^10^ Based on the pNDM-HK sequence (HQ451074), we newly designed a primer pair (*trpF*-F, 5’-ATGCCCGCGAAAATCAAGATTTG-3’; and DHA type-R, 5’-CAAAGCCAGTATGCGTACGG-3’) to know the exact truncated *bla*_DHA-1_ sequence in the *E. coli* test isolate (Fig.1E). Using these two primers, one band (734 bp) was detected in the test isolate (Fig.1F). Sequencing data of this band showed that 345 bp (position: 796 to 1140) of *bla*_DHA-1_ sequence were missing at 3’ end. The total sizes of Δ*bla*_DHA-1_ and *bla*_DHA-1_ were 795 bp and 1140 bp, respectively.^9^,^10^ Therefore, the efficient molecular diagnostic method (_large-scale_*bla*Finder) could not detect the Δ*bla*_DHA-1_ gene in the *E. coli* test isolate. Because a truncated *bla* gene does not show any antibiotic resistance, the _large-scale_*bla*Finder has no problem for monitoring the emergence and dissemination of *bla* genes and minimizing the spread of resistant bacteria. Therefore, the truncation of a *bla* gene is an important reason for an efficient molecular diagnostic method not to detect the *bla* gene.

## CONCLUSION

The efficient large-scale *bla* detection method (_large-scale_*bla*Finder) is a useful test to detect *bla* gene types including almost all clinically available genes with perfect specificity and sensitivity, although the method could not detect the Δ*bla*_DHA-1_ gene in the *E. coli* test isolate. That is because a truncated *bla* gene does not show any antibiotic resistance.
